# Building and Rebuilding: The National Public Health Laboratory Systems and Services Before and After the Earthquake and Cholera Epidemic, Haiti, 2009–2015

**DOI:** 10.4269/ajtmh.16-0941

**Published:** 2017-10-18

**Authors:** Frantz Jean Louis, Josiane Buteau, Jacques Boncy, Renette Anselme, Magalie Stanislas, Mary C. Nagel, Stanley Juin, Macarthur Charles, Robert Burris, Eva Antoine, Chunfu Yang, Mireille Kalou, John Vertefeuille, Barbara J. Marston, David W. Lowrance, Varough Deyde

**Affiliations:** 1 Centers for Disease Control and Prevention, Port-au-Prince, Haiti;; 2 National Public Health Laboratory, Government of Haiti, Port-au-Prince, Haiti;; 3 U.S. Agency for International Development, Port-au-Prince, Haiti;; 4 Centers for Disease Control and Prevention, Atlanta, Georgia

## Abstract

Before the 2010 devastating earthquake and cholera outbreak, Haiti’s public health laboratory systems were weak and services were limited. There was no national laboratory strategic plan and only minimal coordination across the laboratory network. Laboratory capacity was further weakened by the destruction of over 25 laboratories and testing sites at the departmental and peripheral levels and the loss of life among the laboratory health-care workers. However, since 2010, tremendous progress has been made in building stronger laboratory infrastructure and training a qualified public health laboratory workforce across the country, allowing for decentralization of access to quality-assured services. Major achievements include development and implementation of a national laboratory strategic plan with a formalized and strengthened laboratory network; introduction of automation of testing to ensure better quality of results and diversify the menu of tests to effectively respond to outbreaks; expansion of molecular testing for tuberculosis, human immunodeficiency virus, malaria, diarrheal and respiratory diseases; establishment of laboratory-based surveillance of epidemic-prone diseases; and improvement of the overall quality of testing. Nonetheless, the progress and gains made remain fragile and require the full ownership and continuous investment from the Haitian government to sustain these successes and achievements.

## INTRODUCTION

Seven years after the devastating 2010 earthquake and more than 6 years after cholera struck Haiti, the nation is well into its reconstruction of the health system.^1–3^ This includes reliable, efficient, and accessible laboratory network, which is central to any robust health system. Public health laboratories play a key role in establishing diagnosis, confirming infections, supporting surveillance and outbreak response, and generating evidence-based data.^4–8^ All of these services are crucial in decision-making at many levels and influence not only the health and well-being of individuals but also the health security and economies of a nation.^9,10^ As a member state of the World Health Organization (WHO) and a partner in the Global Health Security Agenda (GHSA),^11^ Haiti endorses the principles of GHSA by participating in and contributing to prevention, detection, and response efforts to mitigate public health risks, particularly those which have the potential to cross borders and threaten people worldwide. To prevent, detect, and respond to human and animal infectious disease threats, Haiti must strengthen laboratory services, specimen transport systems, biorisk management, and laboratory quality management systems to meet its commitments under the 2005 International Health Regulations.^12^

Before the 2010 earthquake, Haiti was moving toward strengthening of the public health laboratory infrastructure and accordingly, in February 2006, the National Public Health Laboratory (LNSP: French acronym) was inaugurated in Port-au-Prince.^13^ LNSP was identified by the Haitian Ministry of Public Health (MSPP) as the national reference laboratory, with a leadership role in coordinating laboratory activities nationwide.^13^ The strategic role of LNSP was to protect the public against infectious threats through disease surveillance and control, outbreak investigation and response, pathogen identification and confirmation, hygiene and environmental protection, quality assurance and quality control, research in public health, and continuing education and training.^13^

Although the earthquake was devastating, LNSP was not structurally damaged. However, similar to other health and educational institutions in Haiti, the national public health laboratory system suffered from destruction of many laboratories at the departmental and peripheral levels within the epicenter of the earthquake and loss of life among laboratory workers.^14^ Immediately after the earthquake, there was an increase in the U.S. Government’s support to strengthen the laboratory services in Haiti. In addition to the U.S. President’s Emergency Plan for AIDS Relief (PEPFAR) funding, which began in 2004, Haiti received substantial post-earthquake supplemental funds to help with health system recovery and rebuilding efforts.^2,3^ This manuscript summarizes laboratory-strengthening activities and progress made in Haiti, comparing pre- and post-earthquake periods from 2009 to 2015.

## HAITI LABORATORY SYSTEMS AND SERVICES PRE-EARTHQUAKE

Before 2010, in the absence of a national laboratory strategic plan, the Haitian laboratory systems and network were not well defined.^13^ LNSP started to play its role as a reference laboratory, but because of its still nascent capacity, a number of advanced tests were referred to other laboratories, such as the Rodolphe Merieux Laboratory at the GHESKIO Center, a nongovernmental organization, for tuberculosis (TB) and multidrug-resistant TB (MDR-TB) diagnosis.^15^ LNSP activities were mainly geared toward training on human immunodeficiency virus (HIV) and syphilis rapid tests, CD4 counts, and chemistry and hematology tests to support the PEPFAR program. Additionally, the External Quality Assessment (EQA) unit had just started operations, primarily to support and assess the quality of HIV rapid tests in the country through a biannual proficiency testing (PT) program.^16^ The PT program is a periodic check on testing processes and laboratory performance where six unknown samples are sent by LNSP for testing to participating laboratories in the country, and subsequently the results of all laboratories are analyzed, compared with those of the LNSP, and reported back.^16^ The molecular biology section was limited to early infant diagnosis (EID) for HIV, which started in 2009. Sections such as parasitology, serology, and bacteriology were still at an embryonic phase and TB testing was not fully established.

## HAITI LABORATORY SYSTEMS AND SERVICES POST-EARTHQUAKE

### Implementation of a national tiered laboratory system.

Laboratory strategic planning is critical to define direction and identify key priorities for resources allocation to achieve sustainable standards for quality laboratory services. In 2010, Haiti aligned with the Maputo Declaration to finalize its first quinquennial (2010–2015) national strategic plan to identify the needs and streamline goals, priorities, and resources to reinforce the laboratory health system.^13^ In 2014, MSPP approved the updated “Harmonization and Standardization of the Tiered National Laboratory Network” document, which streamlines the structure of the network, the minimum requirements for laboratories, and sets guidelines for infrastructure and testing menus for each tier in the laboratory network (Table 1).^17^ This laboratory network system is in line with the pyramid tiered model outlined in the Maputo Declaration on strengthening laboratory systems, on January 2008.^18^ Accordingly, LNSP is at the top of the pyramid, designed to serve as a reference laboratory, oversee and coordinate the activities at the three levels of services, and provide a wide range of confirmatory tests. There are tertiary laboratories, typically regional/teaching hospital laboratories, where a comprehensive range of laboratory services are provided. Under them are secondary laboratories, usually departmental hospital laboratories, where tests for clinical care and support of several main diseases of public health importance (HIV, TB cholera, and malaria) are offered. At the bottom of the pyramid is the primary level, peripheral public health-care facilities/laboratories, where basic laboratory tests are performed. The tests performed at each level of the laboratory network vary depending on the population served, level of service available, physical infrastructure, electricity, water, road conditions, and the availability and level of trained technical personnel in country.^17^ Finally, the Haiti national laboratory policy document is in final stage of the development; this document will set the legal and regulatory framework for laboratory licensing, certification, and accreditation to improve national quality management system.

**Table 1:** Tiered laboratory system and menu of tests for each tier level

Tests	National Public Health Laboratory	Regional laboratories (*N* = 4)	Departmental laboratories (*N* = 10)	Peripheral laboratories (over 200)
HIV serology	EIA, rapid tests, WB	EIA, rapid tests	Rapid tests	Rapid tests
CD4 cell count	Flow cytometry machine	Flow cytometry machine	Flow cytometry machine	Specimen referral
HIV EID	TNA PCR	Near POC TNA PCR	DBS to ref laboratory	DBS to ref laboratory
HIV viral load	RNA PCR	Near POC RNA PCR	DBS to ref laboratory	DBS to ref laboratory
Chemistry	Low-volume dry chemistry analyzer	Low-volume dry chemistry analyzer	Low-volume dry chemistry analyzer	Low-volume dry chemistry analyzer
Hematology	Low-volume hematology analyzer	Low-volume hematology analyzer	Low-volume hematology analyzer	Low-volume hematology analyzer
TB	Light and fluorescent microscopy; molecular Hain’s; solid and liquid culture and DST (BSL-3); GeneXpert; slides rechecking	Light and fluorescent microscopy; solid culture; GeneXpert	Light and fluorescent microscopy; GeneXpert	Light or fluorescent microscopy
Syphilis	Treponemal and non-treponemal serology test	Treponemal and non-treponemal serology test	Rapid tests	Rapid tests
Parasitology	Microscopy, serology, and RT-PCR	Microscopy and rapid tests	Microscopy and rapid tests	Rapid tests
Bacteriology	Gram stain, culture, and DST	Gram stain, culture, and DST	Gram stain	Gram stain

BSL-3 = biosafety laboratory 3; DBS = dried blood spots; DST = drug-susceptibility test; EIA = enzyme immunoassay; EID = early infant diagnosis; Hain’s = Genotype *Mycobacterium tuberculosis* Drug Resistance; PCR = polymerase chain reaction; POC = point of care; TNA = total nucleic acid; WB = Western blot.

### Building of the national public health laboratory technical capacity.

**Figure 1.**
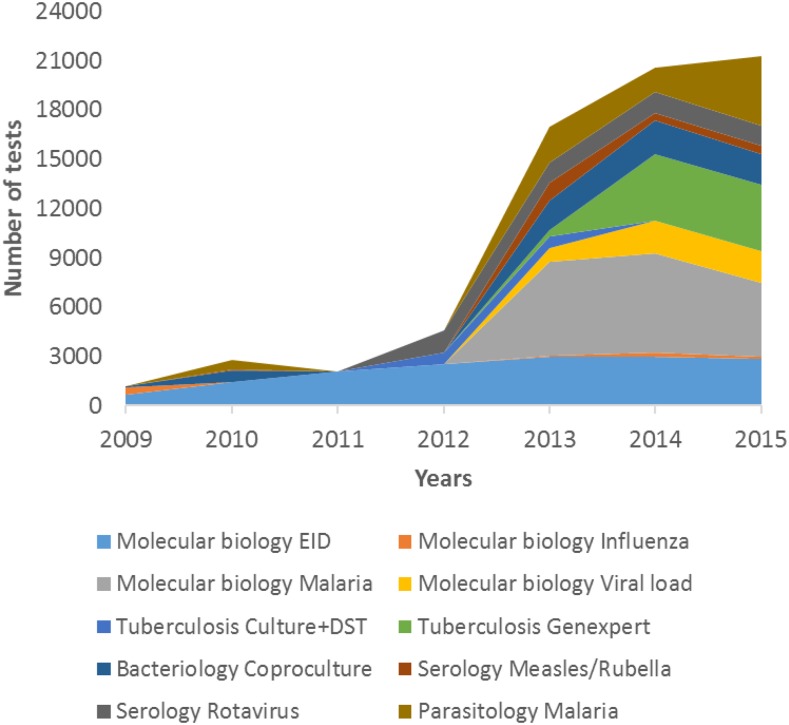
Significant improvement in national public health laboratory testing capacity after rebuilding of infrastructure. Bacteriology testing capacity was limited and focused only on detection of *Salmonella* and *Shigella* for coproculture. Molecular testing was limited to two or three pathogens. Following the rebuilding efforts, culture and molecular diagnostic capacity in the areas of human immunodeficiency virus, tuberculosis, malaria, bacteriology, and other pathogens was expanded.

 With additional funds to support the post-earthquake recovery and rebuilding efforts, LNSP rapidly expanded both the number of laboratory health-care workers and the testing categories to better play its role as a reference laboratory.^2,16,19^ Before 2010, the bacteriology section at LNSP had a limited testing capacity, testing menus was mainly focused on detection of *Salmonella* and *Shigella* for coproculture; however, despite that, the newly established bacteriology section was able to successfully isolate and identify the first *Vibrio cholerae* in Haiti soon after the cholera outbreak.^20^ A couple of months after the start of the cholera epidemic, the number of coproculture increased drastically to assist in the investigation and response to the outbreaks, with more than 75% (*N* = 564) of the samples being identified as having *V. cholera*.^20^ All positive samples were sent to Centers for Disease Control and Prevention (CDC), Atlanta, for confirmation and drug susceptibility testing. The other sections at LNSP followed the same trend after 2010, as the number of EID tests quadrupled to average ∼2,500 children diagnosed every year (Figure 1).^21^ The number of tests performed at LNSP decreased in 2011 as a consequence of the 2010 earthquake and the time it took the Laboratory to regain full capacity of its activities (Figure 1). HIV viral load testing and GeneXpert for TB diagnosis were introduced for the first time to LNSP in 2013 to support HIV and TB care and treatment programs (unpublished data) (Figure 1). In 2014, a temporary modular biosafety laboratory 3 was inaugurated at LNSP to support MDR-TB surveillance in Haiti, pending the completion of a permanent TB laboratory, which is expected to be fully operational by end of 2017. Of note, this temporary capacity was not fully realized due to the destruction of the modular laboratory by fire. Another decrease in testing activities can be observed in 2015; this decrease is attributed to internal restructuring at LNSP, which resulted in major stock out of essential reagents for testing.

### Setup of a laboratory-based system surveillance and response system.

To complement the national syndromic surveillance system, a laboratory-enhanced sentinel surveillance (French acronym: PRESEpi) was launched in April 2012.^22^ PRESEpi was initially designed to monitor conditions associated with two syndromes: acute diarrheal illness (cholera, *Shigella*, and *Salmonella*) and acute febrile illness (malaria, dengue, *Salmonella typhi*, and leptospirosis) at four sentinel hospitals in Haiti. PRESEpi expanded over the years to include two additional sites and surveillance for five additional pathogens.^23^ Surveillance for acute respiratory infections (influenza in particular), meningitis, and rotavirus was introduced at LNSP through this system in 2013 and was aimed at estimating the impact and effectiveness of rotavirus and pneumococcal vaccines introduced in Haiti in 2014. In response to recent outbreaks of arboviruses (chikungunya in 2014 and Zika in early 2016), LNSP was able to confirm the spread of chikungunya in all 10 administrative departments (Boncy J, personal communication, 2014) of the country and is playing a key role in supporting outbreak investigations and responses to the Zika virus epidemic in collaboration with the Caribbean Public Health Agency regional laboratories, WHO/Pan-Amarican Health Organization, and the U.S. CDC (unpublished data).

### Increased investment in laboratory infrastructure and equipment.

**Figure 2.**
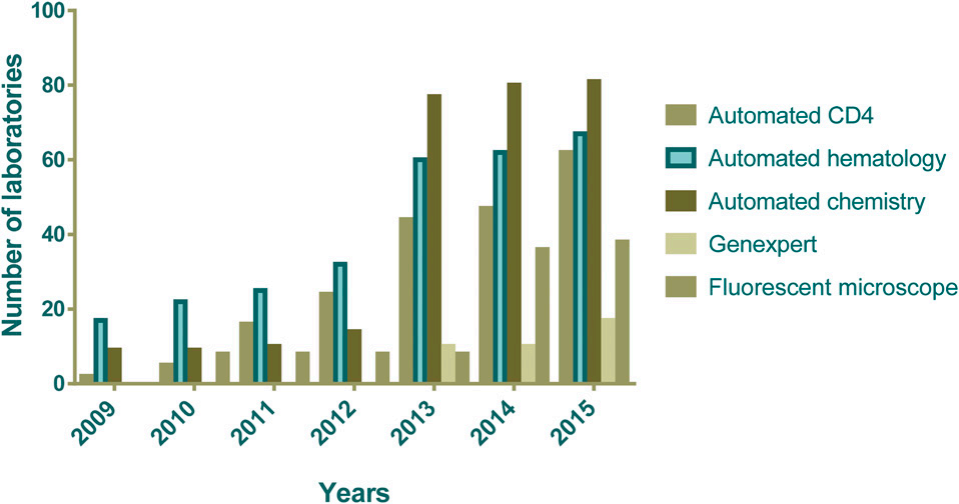
Public health laboratories witnessed tremendous growth with leadership from the Ministry of Health and support from U.S. Government and other partners. The number of laboratories providing automated testing services increased from less than 50 in 2009 to over 200 in 2015. GeneXpert testing for tuberculosis (TB) was introduced in 2013 and is still expanding with tremendous impact on identification and management of TB patients.

**Figure 3.**
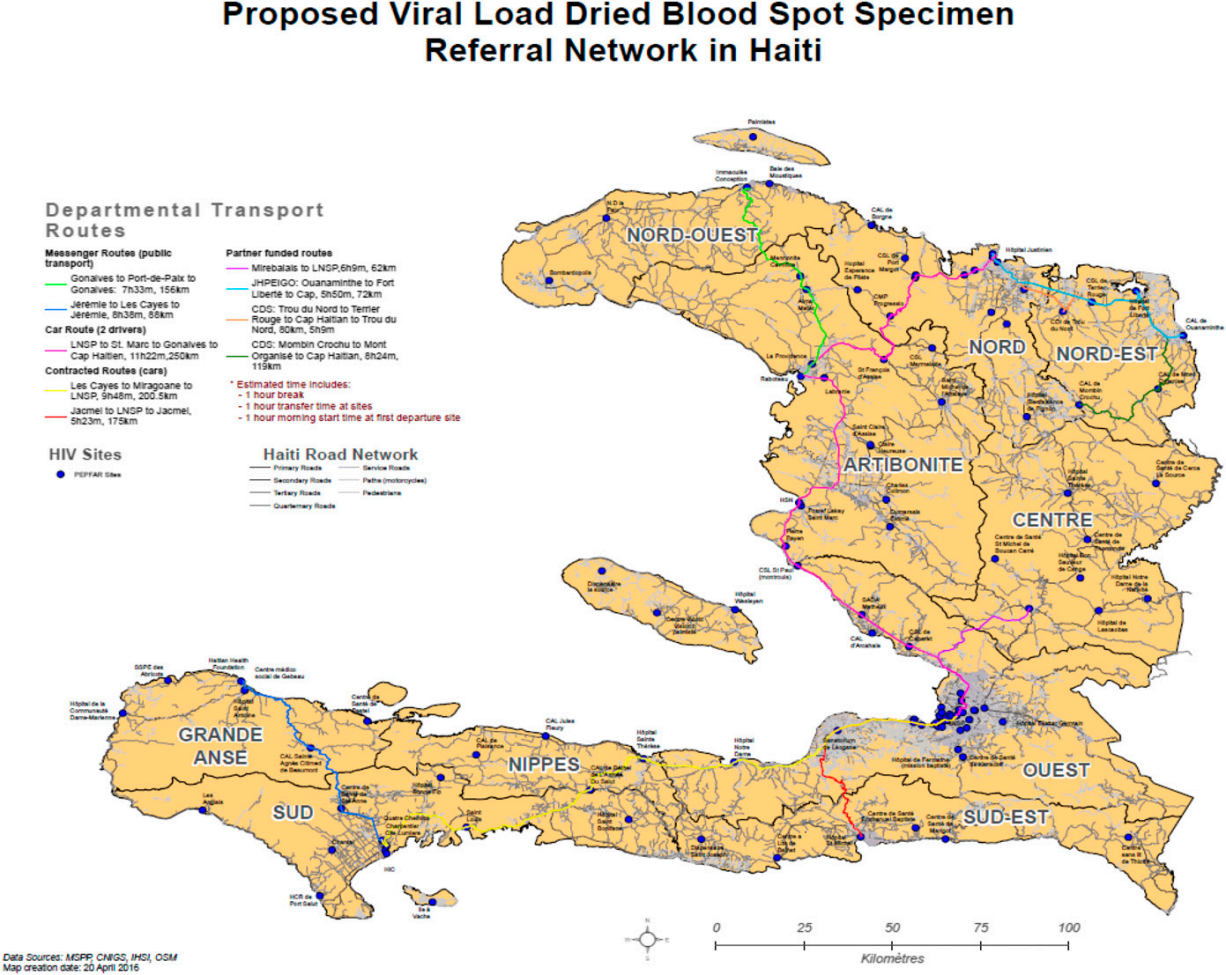
Establishment of a national human immunodeficiency virus (HIV) viral load specimen transport and referral network. In 2015, a national HIV viral load specimen referral network was implemented based on dried blood spots. The program is still expanding to allow access to this service for all people living with HIV to monitor their antiretroviral treatment outcomes. Once completed, this network will serve as a national platform for transport and referral of other specimens collected throughout the country.

Significant investments have been made to repair and renovate the laboratory infrastructure affected by the 2010 earthquake in Haiti.^1^ In addition to funding from the U.S. Government, a tripartite agreement between the governments of Haiti, Cuba, and Brazil assisted in building two regional laboratories (the northern regional laboratory at Justinien Hospital in Cap Haitian and the southern regional laboratory at Immaculée Conception Hospital in Cayes).^24^ Partners in Health is finalizing the construction of the Central Plateau regional laboratory at Mirebalais Hospital to support the decentralization of laboratory capacity in Haiti.^25^ The U.S. Government invested heavily to equip laboratories at all tier levels to support diagnostic services for HIV, TB, and malaria, among other diseases, in Haiti. Automation in CD4 testing completely replaced manual CD4 by the end of 2013 to increase laboratory access to reach 99% of people living with HIV/AIDS through a spoke-and-hub specimen referral system by 2014 (Figure 2); before 2013, an average of only 20% of health-care facilities had access to CD4 testing.^15^ Automation in chemistry and hematology testing expanded until 2013 and stabilized to provide better quality of testing services and GeneXpert platforms became available at 17 geographically distributed laboratories by 2015 to strengthen TB diagnostics (Figure 2). Moreover, in 2015, LNSP developed a national specimen referral network for HIV viral load testing using dried blood spots (DBSs), with support from PEPFAR and CDC non-PEPFAR funds (Figure 3). Of note, Haiti has adopted the WHO recommendation to use viral load testing for treatment monitoring and is phasing out the use of CD4 tesing.^21^ Successful implementation of the DBS national specimen referral network will allow incorporation of other specimen transport systems and streamline efforts toward an integrated, efficient, and sustainable specimen transport program at the national level.

### Expansion of laboratory quality assurance services.

**Figure 4.**
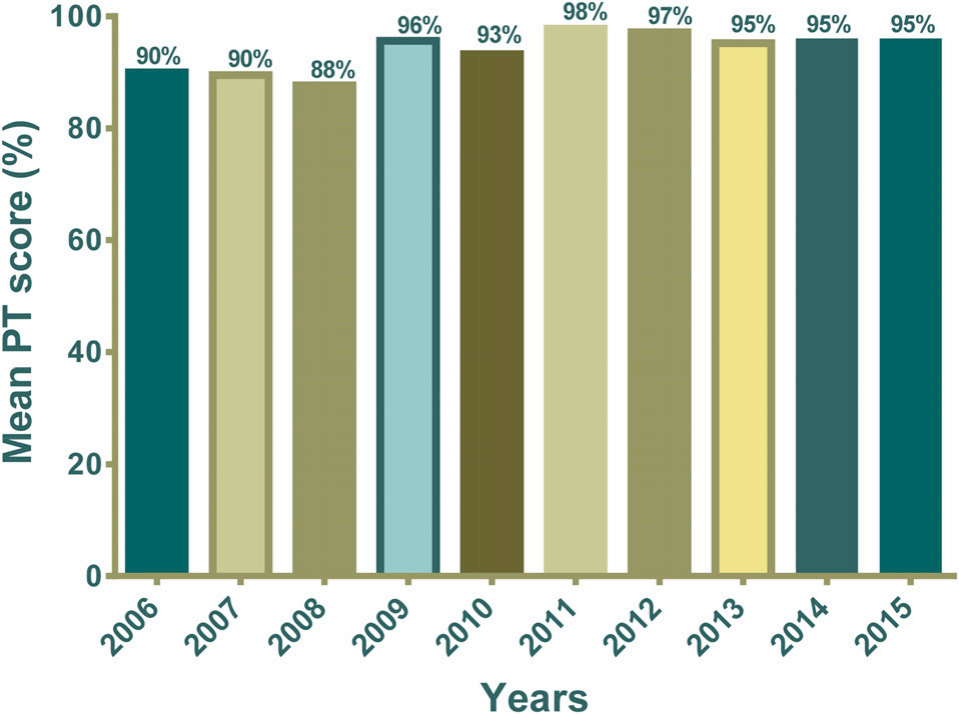
Improved public health laboratory services as measured by performance of laboratories participating in proficiency testing (PT) schemes. Prior to the earthquake, the average passing scores of participating laboratories were around 90% and were improved from 95% up to 98% after earthquake.

In 2012, LNSP and the regional laboratories enrolled in the WHO-Afro Strengthening Laboratory Management Towards Accreditation (SLMTA) program^26^ and successfully completed three interactive workshops. Certified SLMTA assessors conducted pre- and post-SLMTA assessments to measure the improvement following SLMTA trainings. The assessment findings indicated that LNSP improved from 0 to 1 star, based on defined quantitative set of indicators to measure quality assurance.^27^ In addition, LNSP partners with the College of American Pathologists for EQA for their parasitology, bacteriology, and immunology sections. For TB and molecular biology (HIV PCR and EID), LNSP receives panels twice a year from CDC, Atlanta, for continuous assessment of quality improvements. Since its creation in 2006, the EQA program has been a priority for LNSP to ensure timely, reliable, and accurate laboratory testing services.^16^ The biannual rapid test EQA program enrolled laboratorians from all PEPFAR-supported institutions except for private laboratories to systematically evaluate the quality of HIV rapid testing. The results of the HIV rapid test EQA program from 2006 to 2015 showed national scores consistently over 95% after 2010 compared with an average score of 91.4 before the earthquake (Figure 4).^16^ The national PT score decreased from 98% to 95% after 2011; this can be explained by the national effort to expand access to HIV care and treatment by increasing the number of sites providing HIV RDT across the country. These newly enrolled laboratories tend to perform lower than the already established ones. In addition, high staff turnover affects the EQA results, reinforcing the need for continuous training and onsite technical assistance. Participation in the HIV EQA program has been robust over the years with an average of 92% compliance of laboratories enrolled to submit the EQA panels to LNSP.^16^ Annual PT panels were also distributed to all PEPFAR-supported institutions to assess syphilis rapid tests, CD4 count, chemistry, hematology, and GeneXpert. Acceptable scores (over 83%, which is 5 of 6 unknown samples correctly identified) were observed from the majority of participating laboratories.^16^ For participating laboratories with poor performance, corrective action plans were developed and implemented, and onsite technical assistance and retraining were conducted, which significantly improved their PT scores as shown in previously published data.^16^ Slide rechecking was conducted to assess the accuracy of malaria and TB diagnostics using microscopy with acceptable performance from all participating laboratories.^16^

## CHALLENGES AND OPPORTUNITIES

Beside the significant progress made by LNSP and the national laboratory network to support disease detection, improve surveillance, and effectively respond to outbreaks, many challenges remain. A 5-year National Strategic Plan 2010–2015 was developed and endorsed by MSPP in 2010 streamlining the core elements that need to be strengthened to reinforce the national laboratory network.^13^ In line with the new priorities of the laboratory system in Haiti and its engagement with the GHSA, a new strategic plan needs to be developed to strengthen laboratory-based surveillance. For LNSP to fully play its role as a reference public health laboratory, significant improvements in quality assurance are needed and efforts to achieve higher scores in quality improvements with the ultimate goal to reach accreditation should be prioritized.

Like in many other resource-limited countries, laboratory equipment maintenance and repair is another challenge for the laboratory system in Haiti.^28^ Currently, equipment repair has to be outsourced through expensive and unsustainable contracts, because of the unavailability of trained biomedical engineers in country.

The nearly total dependence of the laboratory system, including basic and core services, on external funding (mainly from the U.S. Government through PEPFAR and post-earthquake supplemental funds) is a threat to a sustainable public health system that needs to be addressed.

Qualified human resources are also a key component for any successful laboratory network system and this capacity needs to be strengthened in Haiti. A new curriculum was developed in 2013 to upgrade the national laboratory schools from a 2-year laboratory technician teaching program to deliver a 4-year bachelor degree; however, resource limitations have hampered implementation of the program. In 2011, the Merieux Foundation, in collaboration with LNSP and the Catholic University of Lyon, France, implemented a postgraduate program for in-service laboratory technicians to upgrade their training to a bachelor degree level.^29^ The development and implementation of a national laboratory policy to regulate both public and private laboratories in the country will improve provision of laboratory services and limit delays or compromising treatment. Furthermore, the current efforts aimed at developing and implementing a national laboratory information system would provide an opportunity to link laboratory data to the national surveillance reporting platforms and improve the availability of these data for timely decision-making.

## CONCLUSIONS

Tremendous progress has been made in the area of laboratory systems and services, and the current laboratory service platforms’ technical expertise provide a real opportunity for the MSPP and LNSP to play leading roles as centers of excellence in the region. With the support of the U.S. Government, the Haitian laboratory system is on its way to recovery after the devastating 2010 earthquake and cholera outbreak. Significant progress has been made in building stronger laboratory infrastructure and a more qualified public health laboratory workforce across the country to decentralize access to quality-assured services, automate testing to ensure better quality of results, and diversify the menu of tests to effectively respond to outbreaks. Increased investment from the Haitian government to sustain the gains made will ensure continued improvements in the laboratory network in Haiti.
